# The effect of brisk walking in the fasted versus fed state on metabolic responses, gastrointestinal function, and appetite in healthy men

**DOI:** 10.1038/s41366-018-0215-x

**Published:** 2018-09-24

**Authors:** Victoria J. McIver, Lewis Mattin, Gethin H. Evans, Adora M. W. Yau

**Affiliations:** School of Healthcare Science, Manchester Metropolitan University, Manchester, M1 5GD UK

**Keywords:** Metabolism, Endocrinology

## Abstract

**Objective:**

To investigate the effect of brisk walking in the fasted versus fed state on gastric emptying rate (GER), metabolic responses and appetite hormone responses.

**Subjects/methods:**

Twelve healthy men completed two 45 min treadmill walks, fasted (FASTED) and followed consumption of a standardised breakfast (FED). GER of a standardised lunch was subsequently measured for 2 h using the ^13^C-breath test method. Blood samples were collected at baseline, post-breakfast period, pre-exercise, immediately post exercise, pre-lunch then every 30 min following lunch for 2 h. Circulating concentrations of acylated ghrelin (GHR), glucagon-like peptide-1 (GLP-1), peptide tyrosine tyrosine (PYY), pancreatic polypeptide (PP), glucose, insulin, triglycerides, non-esterified fatty acids (NEFA) and cholesterol were measured. Subjective feelings of appetite were assessed at 15 min intervals throughout. Substrate utilisation was measured every 30 min, and continuously throughout exercise by indirect calorimetry.

**Results:**

No differences were observed for GER T_½_ (FASTED 89 ± 22 vs. FED 89 ± 24 min, *P* = 0.868) nor T_lag_ (FASTED 55 ± 15 vs. FED 54 ± 14 min, *P* = 0.704). NEFA concentrations were higher in FASTED at pre-exercise, post exercise and 30 min post exercise (pre-lunch) (all *P* < 0.05) but no differences were observed for glucose, cholesterol or triglycerides. Carbohydrate oxidation was greater at all time-points during FED exercise (all *P* < 0.05). Minimal changes in appetite were observed post lunch ingestion with no differences in PYY or GHR observed between trials. GLP-1 concentrations were greater in FED post-breakfast and pre-exercise (*P* < 0.05), though no differences were observed after lunch. A greater concentration of PP was observed in FED from pre-exercise to 30 min post lunch consumption (all *P* < 0.05). Insulin concentrations were higher in FED pre-exercise but higher in FASTED 1.5 h post lunch (*P* < 0.05).

**Conclusion:**

These findings suggest that gastrointestinal function, hunger and appetite regulatory hormones are not sensitive to low-intensity bouts of physical activity and holds positive implications for weight management practices.

## Introduction

With the prevalence of obesity reaching epidemic proportions worldwide, it remains a significant economic burden to healthcare systems globally. Intermittent fasting has been suggested as an effective method to promote health and prevent the onset of metabolic diseases by increasing insulin sensitivity and fatty-acid mobilisation, and reducing inflammation [1]. In addition, exercise alone induces health benefits, preventing the onset of metabolic diseases by modulating a range of risk factors [2–8]. Interest in the effect of fasted exercise has grown recently in both the scientific field [9–12] and the general public. However, the efficacy of whether exercising in the fasted state compared with the postprandial state is more beneficial for reducing energy intake and inducing a negative energy balance for weight loss remains unclear.

The regulation of appetite and gastrointestinal motility appears to be intrinsically linked as the rate of gastric emptying determines the time of gastric distention, which is known to be a satiety signal [13, 14]. Therefore, the effect of fasted exercise on gastric emptying rate (GER) may be an important mechanistic consideration for subsequent food and energy intake. GER may also play a role in metabolic health as the delivery and absorption of nutrients in the small intestine is largely dependent on this process. Consequently, the influence of fasted exercise on gastrointestinal function is also of interest in postprandial metabolic responses. A previous study has shown postprandial glucose response to be less following fasted exercise [15]. This could be due in part to differences in GER. To the authors knowledge, no studies have investigated the effects of fasted exercise compared with fed exercise on gastrointestinal function and the consequence this may have on metabolic responses.

GER is influenced by a number of gut-derived hormones. The orexigenic hormone ghrelin (GHR) has been shown to increase GER [16, 17], whereas satiety hormones such as peptide tyrosin tyrosin (PYY), glucagon-like peptide-1 (GLP-1) and cholescystokinin have been shown to decrease GER [18–22]. Research investigating the effect of exercise interventions on appetite regulation and energy intake have tended to adopt the conventional method of participants' exercising following an overnight fast with no comparative postprandial condition [23–30]. The majority of these studies have reported that an acute bout of exercise reduces subjective ratings of hunger and alters appetite-regulating hormones, decreasing the orexigenic hormone GHR, and increasing the satiety hormones peptide YY and leptin for a period of time post exercise. It is currently unknown whether exercising in the fasted state results in different gut hormone responses to exercising in the postprandial state. Comparing the effect of an acute bout of fasted or fed exercise on appetite regulatory hormone responses may provide knowledge on the mechanisms relating to gastrointestinal function responses and appetite regulation that could potentially lead to intervention strategies or advice for individuals aiming to achieve a negative energy balance for weight loss. Studies that have measured gut hormone responses to fasted and postprandial exercise have reported fasted exercise increased GHR concentrations and fed exercise tended to elevate PYY concentration [31], whereas another observed no differences between conditions for GLP-1 (7–36) and insulin responses [15]. Furthermore, studies that have measured gut-derived appetite hormones in response to exercise have tended to measure only one or two hormones, with GHR, GLP-1 and PYY being the most common [23–30, 32]. Simultaneous measurement of these and other gut-derived appetite hormones would provide a clearer picture of the effects of exercise on appetite regulation.

Investigations on the effect of fasted versus non-fasted exercise interventions on appetite regulation and energy intake have reported fasted exercise reduces appetite [31, 32] and 24 h energy intake [12]. In addition, fasted exercise has been shown to increase fat oxidation [12] and provides beneficial metabolic adaptations [33]. On the other hand, some studies have found no differences between the effects of fasted or non-fasted exercise, with both inducing weight loss [34] and improving metabolic responses and body composition [10, 34]. However, these studies have typically used a high-fat (70%) meal [31], which is not representative of a typical breakfast, compared meal-exercise sequence rather than omission of breakfast per se [35], or have not measured appetite hormone responses [33, 34]. In addition, these studies have commonly used highly trained populations and/or adopted exercise that is of higher intensity or that is reliant on the use of exercise equipment, thus, lacking exercise that can be accessible to all. These factors make it difficult to generalise the results to the wider or clinical populations and studies are required to investigate the effect of fasted exercise on appetite regulation in these populations. Brisk walking is the most popular modality of physical activity undertaken on a general population level [36], owing to it being easily accessible and with no requirement for specialised equipment. Thus, investigating whether fasted brisk walking may have more favourable benefits on appetite regulation compared with non fasted walking is of interest.

The aim of this study was to investigate the effect of brisk walking in the fasted versus fed state, on GER, and associated metabolic and appetite hormone responses. It was hypothesised (a) that GER would be different between fasted and fed exercise (b) that fasted and fed exercise would result in different appetite and metabolic responses and (c) that these differences would be mediated by respective responses in appetite hormones.

## Materials and methods

### Participants

Twelve recreationally active men (Mean ± SD; age 26 ± 5 years; height 179 ± 6 cm; body mass 86 ± 14 kg; body mass index (BMI) 27 ± 4 kg/m^2^; V̇O_2peak_ 39 ± 6 ml/kg/min) volunteered to participate in this study. Participant number was determined by a power analysis based on data that would result in a detectable change in GER and fat oxidation with 80% power and at a significance level of 5%. Participants were not taking regular medication or with any known history of respiratory, cardiovascular or chronic gastrointestinal disease as assessed by a health-screen questionnaire. All participants were also free from musculoskeletal injury and were non-smokers. All participants were informed of the details of the study both verbally and in writing prior to providing their written informed consent. The study was approved by the Faculty Ethics Committee (Reference: SE161749).

### Preliminary trial

All participants attended a preliminary trial at least 7 days prior to the first experimental trial. This visit involved the collection of anthropometric measures as well as familiarisation of breath sampling procedures. Height was measured to the nearest 0.1 cm using a wall-mounted stadiometer and body mass to the nearest 0.01 kg using electronic scales (GFK 150; Adam Equipment Co. Ltd., Milton Keynes, UK). Body fat percentage was approximated using bioelectrical impedance analysis (Omron BF306; Kyoto, Japan).

Following this, all participants completed a peak oxygen uptake (V̇O_2peak_) test on a motorised treadmill. Initially, the treadmill speed was adjusted until a suitable brisk walking pace was determined. Participants were advised that brisk walking is defined as an exercise intensity yielding a mild shortening of breath yet still enabling to converse. Participants maintained this speed for 5 mins. The speed of the treadmill was then increased to 8–12 km/h and the gradient increased by 2.5% every 3 min until volitional exhaustion. Expired air was continuously collected using a breath by breath gas analyser (Metalyzer 3b, Cortex, Leipzig, Germany) and V̇O_2peak_ was calculated by averaging the highest oxygen volume consumed over the final 1 min period. Heart rate was measured continuously using a heart rate monitor (Polar H7, Kempele, Finland) and participants rating of perceived exertion (RPE) [37] was recorded every 3 min.

Before leaving the laboratory, participants were provided with food weighing scales and asked to record their physical activity and food intake in the 24 h before the start of their first experimental trial. Participants were then asked to replicate their activity and diet the day preceding their subsequent trial. Participants were requested to refrain from alcohol consumption, strenuous exercise and caffeine ingestion 24 h before trials.

### Experimental trials

Participants completed two experimental trials in a randomised crossover fashion. Each trial commenced in the morning between 0800 and 0900 h and trials were separated by at least 7 days.

Participants were required to fast from 2000 h the night before experimental trials with the exception of plain water consumption and consumed 500 ml of water 90 min before arrival. Upon arrival to the laboratory, participants were asked to empty their bladder before body mass was recorded. Baseline assessments of appetite (hunger, fullness, prospective food consumption and satisfaction) were made using 100 mm visual analogue scales (VAS) [38], and expired air samples collected for 10 min for the calculation of substrate utilisation. The average V̇O_2_ and V̇CO_2_ measurements from the last 5 min of expired air collection was used to calculate fat and carbohydrate oxidation rates using stoichiometric equations [39]. This sampling method for expired air was adhered to for all resting expired air samples throughout.

Following baseline measurements, participants ate the test breakfast (FED) or remained fasted (FASTED) within a 15 min period. The breakfast consisted of 30 g of breakfast cereal with 125 ml of semi-skimmed milk. This amount was chosen based on the manufacturer’s recommendation of an average serving and provided 733 kJ (175 kcal), 2.75 g fat, 30 g carbohydrate and 7.2 g protein. Participants consumed all of the breakfast within the 15 min window. Post breakfast ratings of appetite and substrate utilisation were measured at the end of the 15 min breakfast period. Participants rested for 1 h before commencement of the exercise protocol. During this 1 h rest period, further measures of appetite were taken every 15 min and substrate utilisation every 30 min.

**Fig. 1:**
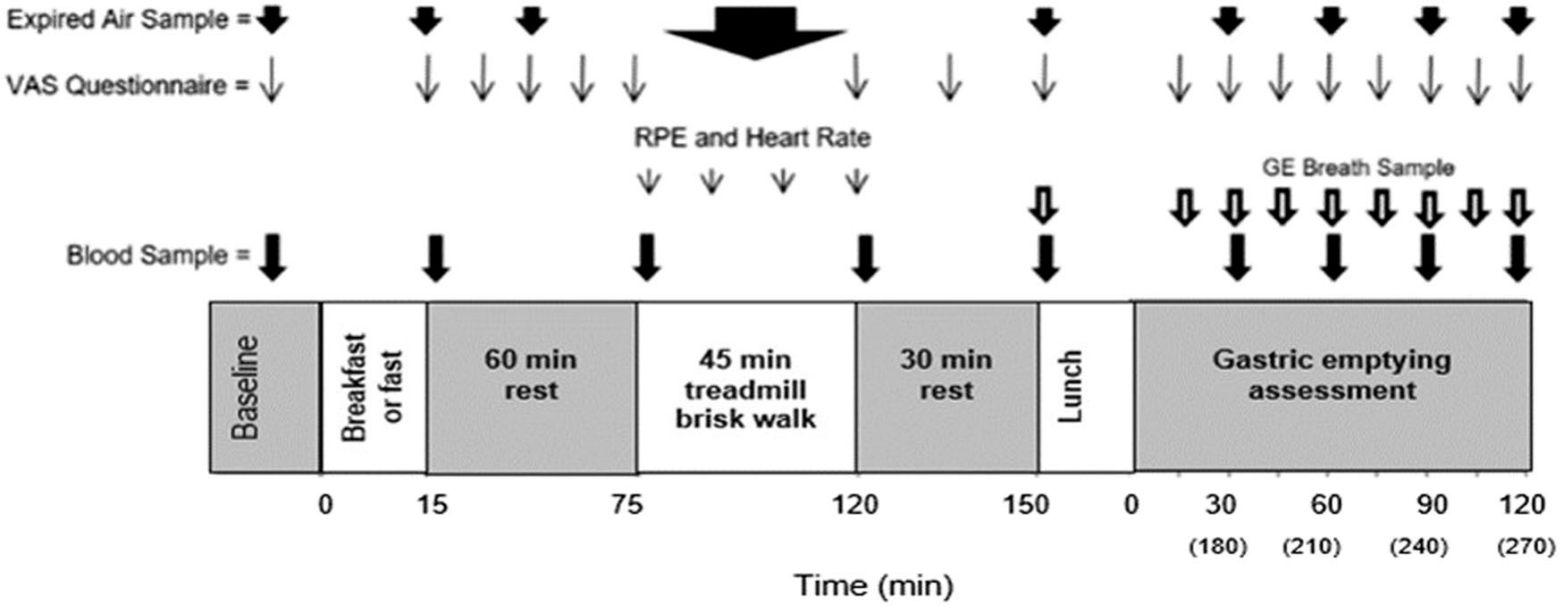
Schematic diagram of the experimental trial protocol. RPE, rating of perceived exertion. GE, gastric emptying

The exercise protocol involved 45 min of brisk walking on a level motorised treadmill at the speed determined in the preliminary trial (range 5.7–6.6 km/h). The relative exercise intensity was 50 ± 0.8% VO_2peak_. Heart rate and RPE were measured every 15 min throughout the exercise, with expired air measured continuously. The last 10 min of each 15 min segment was used to calculate substrate utilisation. Participants recovered for 30 min before they ingested a standardised meal. The meal was 400 g (one can) of chicken and sweetcorn soup (1013 kJ (242 kcal)), containing 11.8 g fat, 25.1 g carbohydrate, 8.2 g protein. Subjective feelings of appetite and substrate utilisation were measured every 15 min post exercise for a total period of 2 h. A schematic diagram of the experimental protocol is presented in Fig. 1.

### Blood sampling and blood sample analysis

An intravenous cannula was inserted into an antecubital vein, which remained in place for the duration of the trial. The cannula was kept patent with the infusion of isotonic saline after each sample collection. Blood samples were collected at baseline, post breakfast period, pre-exercise, immediately post exercise, pre-soup ingestion, then every 30 min post soup ingestion. To prevent the degradation of acylated GHR and active GLP-1, 50 µl of Pefabloc (Roche Diagnostics Limited, Burgess Hill, UK) and 50 µl of dipeptidyl peptidase IV inhibitor (Merck Millipore Ltd., Feltham, UK) was immediately added to blood samples. Blood samples were centrifuged at 1500 × *g* for 15 min at 4 °C and the serum aliquoted and stored at − 80 °C until analysis. Serum glucose, non-esterified fatty acids (NEFA), triglycerides and total cholesterol concentrations were determined in duplicate using a clinical chemistry analyser (Randox Daytona, Crumlin, UK). Circulating concentrations of acylated GHR, active GLP-1 (7–36 and 7–37 forms), total PYY, pancreatic polypeptide (PP), and insulin were determined using multiplex analysis (Luminex 200, Luminex Corporation, Austin, TX, USA) with kits purchased from Merck Millipore (HMHMAG-34K, Milliplex MAP, Merck Millipore Ltd., Feltham, UK).

### Gastric emptying assessment

The soup contained 100 mg of ^13^C-sodium acetate for the assessment of GER using the ^13^C-breath test method. A basal end-expiratory breath sample was collected pre-meal ingestion then at every 15 min intervals post meal ingestion for 2 h. Breath samples were analysed for the ratio of ^13^CO_2_:^12^CO_2_ by non-dispersive infra-red spectroscopy (IRIS Dynamic, Kibion, Germany). The difference in the ratio of ^13^CO_2_:^12^CO_2_ from baseline breath to post-ingestion breath samples are expressed as delta over baseline (DOB). Half-emptying time (T_½_) and time of maximum emptying rate (T_lag_) were calculated utilising the manufacturers integrated software evaluation incorporating equations of a previously described formula [40].

### Statistical analysis

A two-way repeated measures analysis of variance (ANOVA) was used to assess trial × time differences in serum blood measures gastric emptying DOB, substrate oxidation and VAS ratings. Sphericity for repeated measures was assessed, and where appropriate, Greenhouse–Geisser corrections were applied for epsilon < 0.75, and the Huynh–Feldt correction adopted for less-severe asphericity. Significant *F* tests were followed by dependent Student’s *t* tests or one-way repeated ANOVA and Bonferroni adjusted pairwise comparisons as appropriate. Gastric emptying T_½_ and T_lag_ data were analysed using dependent Student’s *t* test. All analyses were carried out using IBM SPSS statistics (v22.0 for Windows; SPSS, Chicago, IL). The level of significance was set at *P* < 0.05. Descriptive data are expressed as mean ± standard deviation (SD).

## Results

### GER

**Fig. 2:**
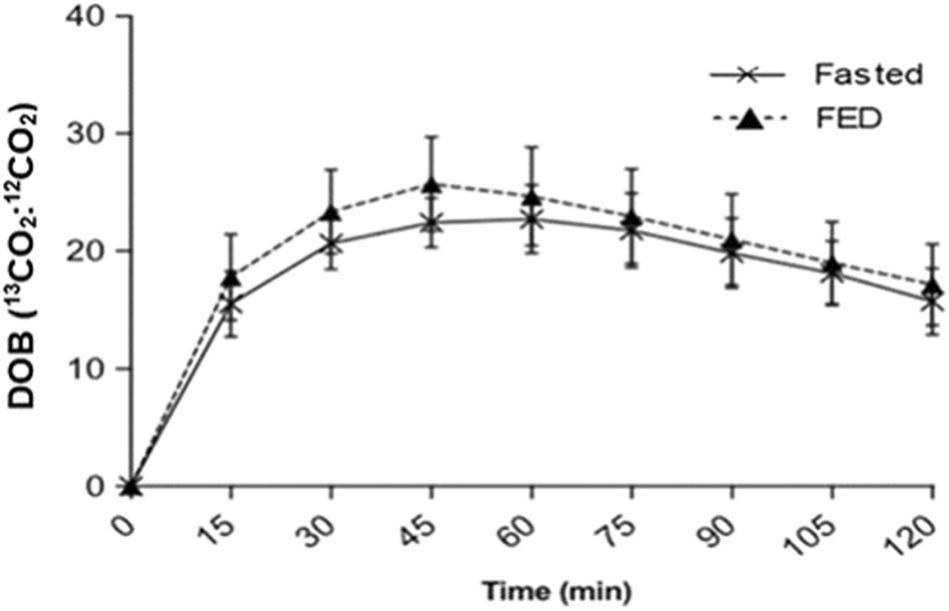
Gastric emptying delta over baseline (DOB) of the lunch provided (400 g chicken and sweetcorn soup) following 45 min of brisk walking either fasted (FASTED) or after breakfast consumption (FED). Values were mean ± SD; *n* = 12

No differences between trials (FASTED v. FED) were observed for gastric emptying T_lag_ (55 ± 15 vs. 54 ± 14 min; *P* = 0.704), and T_½_ (89 ± 22 vs. 89 ± 24 min; *P* = 0.868). No trial × time interaction effect (*P* = 0.341) or main effect of trial (*P* = 0.332) was detected for DOB, although, a main effect for time was found (*P* < 0.001) (Fig. 2).

### Subjective feelings of appetite

**Fig. 3:**
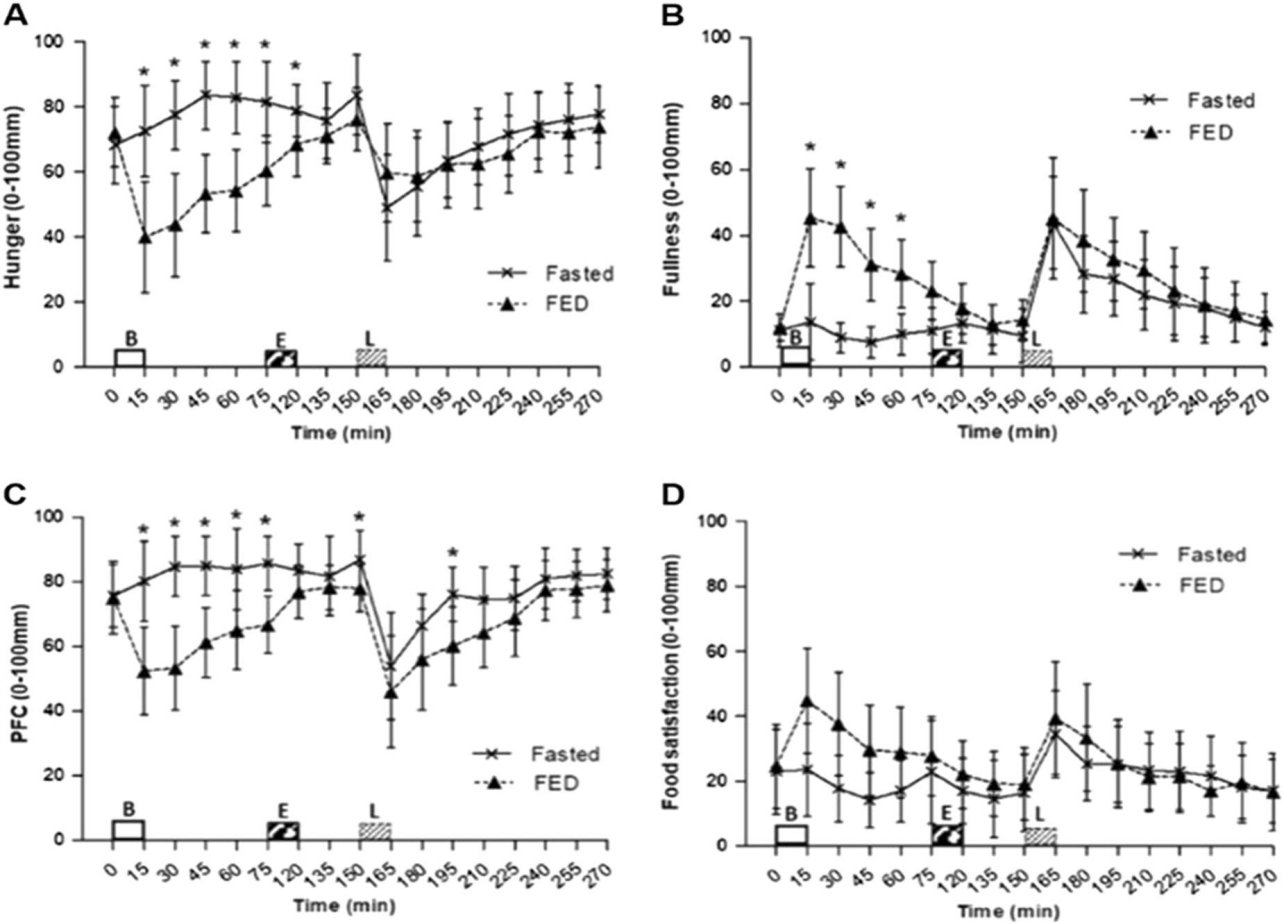
Appetite scores during trials, assessed by 100 mm visual analogue scale (VAS); **a** hunger, **b** fullness, **c** prospective food consumption (PFC) and **d** food satisfaction. Values represent mean ± 95% CIs; *n* = 12. *P < 0.05 versus corresponding time point in other trial. B = Breakfast period, in which participants ingested a prescribed breakfast during the FED trial and remained fasted during the FASTED trial, *E* = Exercise period, where participants completed a 45 min brisk walk, *L* = Lunch where 400 g chicken and sweetcorn soup was ingested

A main effect of trial (*P* = 0.006), time (*P* = 0.006) and trial × time interaction effect (*P* < 0.001) was observed for hunger. Hunger ratings were lower post breakfast consumption in FED compared with FASTED at 15 min (40 ± 27 vs. 73 ± 22 mm, *P* = 0.004), 30 min (44 ± 25 vs. 78 ± 17 mm, *P* < 0.001), 45 min (53 ± 19 vs. 84 ± 17 mm, *P* < 0.001), 60 min (54 ± 20 vs. 83 ± 18 mm, *P* < 0.001) and 75 min (60 ± 17 vs. 81 ± 20 mm, *P* = 0.001). This remained lower immediately post exercise (68 ± 15 vs. 79 ± 13 mm, *P* = 0.024), however, no further differences were found following this (Fig. 3a).

A main effect of trial (*P* = 0.008), time (*P* < 0.001) and trial × time interaction effect (*P* < 0.001) was observed for fullness. Fullness was higher post breakfast consumption at 15 min (45 ± 24 vs. 14 ± 18 mm, *P* = 0.008), 30 min (43 ± 19 vs. 9 ± 7 mm, *P* < 0.001), 45 min (31 ± 17 vs. 8 ± 7 mm, *P* < 0.001) and 60 min (28 ± 16 vs. 10 ± 10 mm, *P* = 0.007) in FED compared with FASTED (Fig. 3b).

A main effect of trial (*P* < 0.001), time (*P* < 0.001) and trial × time interaction effect (*P* < 0.001) was observed for prospective food consumption. A higher prospective food consumption was seen post breakfast period at 15 min (80 ± 20 vs.52 ± 22 mm, *P* = 0.004), 30 min (85 ± 15 vs. 53 ± 20 mm, *P* < 0.001), 45 min (85 ± 14 vs.61 ± 17 mm, *P* < 0.001), 60 min (84 ± 20 vs. 65 ± 19 mm, *P* = 0.009), 75 min (86 ± 13 vs.67 ± 14 mm, *P* < 0.001), post exercise prior to lunch at 150 min (87 ± 14 vs. 78 ± 11 mm, *P* = 0.014) and 45 min post lunch ingestion (76 ± 13 vs. 60 ± 19 mm, *P* = 0.009) in FASTED compared with FED (Fig. 3c).

No main effect of trial (*P* = 0.408) or trial × time interaction effect (*P* = 0.149) was observed for food satisfaction. There was a main effect of time (*P* = 0.011), however no differences between trials were found (Fig. 3d).

### Substrate oxidation

**Fig. 4:**
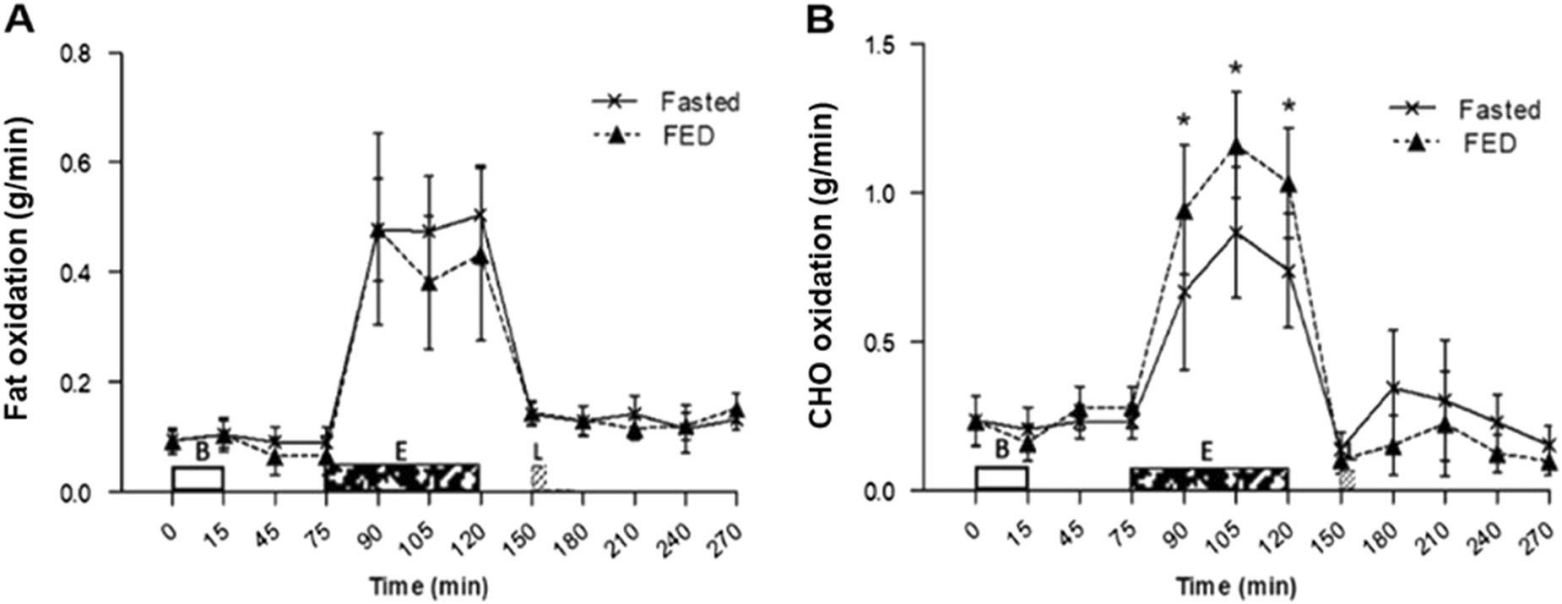
Substrate utilisation throughout both trials. **a** Fat oxidation and **b c**arbohydrate (CHO) oxidation . Values represent mean ± 95% CIs; *n* = 12. *P < 0.05 versus corresponding time point in other trial. *B* = Breakfast period, in which participants ingested a prescribed breakfast during the FED trial and remained fasted during the FASTED trial, E = Exercise period, where participants completed a 45 min brisk walk, *L* = Lunch where 400 g chicken and sweetcorn soup was ingested

No trial × time interaction effect *(P* = 0.096) or main effect of trial (*P* = 0.374) was observed for fat oxidation, although, a main effect of time was present (*P* *<* 0.001) (Fig. 4a). Carbohydrate oxidation also had no main effect of trial (*P* = 0.193). However, a main effect of time (*P* < 0.001), and a trial × time interaction effect (*P* < 0.001) was seen. Carbohydrate oxidation was higher at 15 min (0.94 ± 0.34 vs. 0.67 ± 0.41 g/min; *P* = 0.039), 30 min (1.16 ± 0.28 vs. 0.87 ± 0.35 g/min; *P* = 0.023) and 45 min (1.03 ± 0.29 vs. 0.74 ± 0.30 g/min; *P* = 0.015) of exercise in FED compared with FASTED (Fig. 4b).

### Metabolic markers

**Fig. 5:**
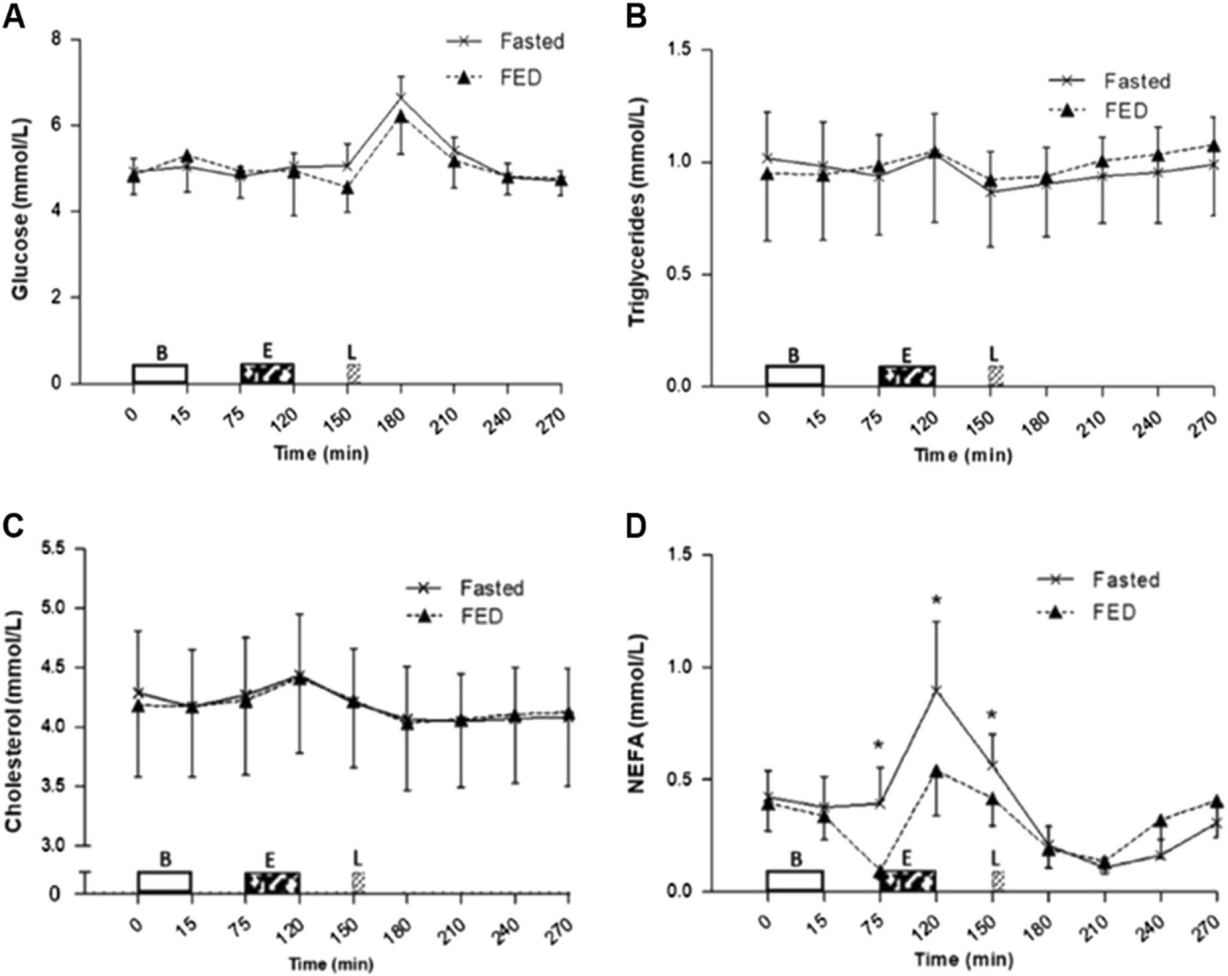
Metabolic responses during trials. Serum concentrations of **a** glucose, **b** triglycerides, **c** cholesterol and **d** non-esterified fatty acids (NEFA). Values represent mean ± 95% CIs; *n* = 12. *P < 0.05 versus corresponding time point in other trial. B = Breakfast period, in which participants ingested a prescribed breakfast during the FED trial and remained fasted during the FASTED trial, E = Exercise period, where participants completed a 45 min brisk walk, *L* = Lunch where 400 g chicken and sweetcorn soup was ingested

No main effects of trial or trial × time interaction effects was seen for blood glucose concentration (*P* = 0.128 and *P* = 0.217; Fig. 5a) and cholesterol concentration (*P* = 0.930 and *P* = 0.383; Fig. 5c). No main effect of trial (*P* = 0.729) but a trial × time interaction effect tending to significance (*P* = 0.056) was seen for triglycerides concentration (Fig. 5b). Main effects of time were seen for blood glucose, triglycerides and cholesterol (all *P* < 0.001).

A main effect of trial (*P* = 0.037), time (*P* < 0.001) and trial × time interaction effect (*P* < 0.001) was seen for NEFA concentration (Fig. 5d). NEFA concentrations in the FASTED trial were greater post breakfast period (pre-exercise) at 75 min (0.39 ± 0.26 vs. 0.09 ± 0.06 mmol/l; *P* = 0.003), immediately post exercise at 120 min (0.90 ± 0.48 vs. 0.54 ± 0.32 mmol/l; *P* = 0.004), and pre-lunch ingestion at 150 min (0.56 ± 0.22 vs. 0.42 ± 0.19 mmol/l; *P* = 0.009). However, NEFA concentrations were higher in the FED trial compared with the FASTED at 1.5 h post soup consumption (0.32 ± 0.27 vs. 0.16 ± 0.11 mmol/l; *P* = 0.018).

### Gut Hormones

**Fig. 6:**
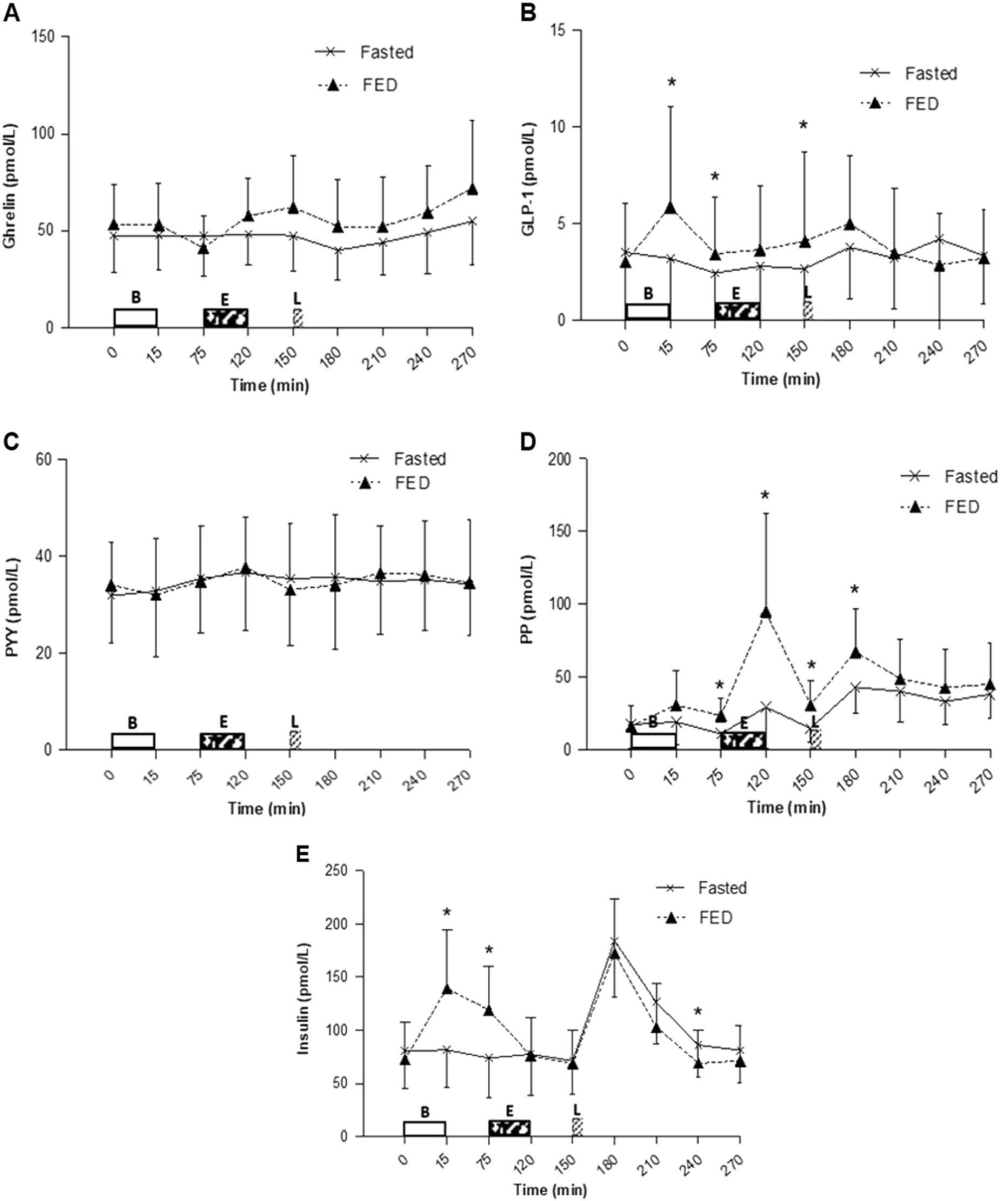
Hormonal responses during both trials. Serum concentrations of **a** ghrelin (*n* = 12), **b** glucagon-like peptide-1 (GLP-1) (*n* = 10), **c** Peptide tyrosin tyrosin (PYY) (*n* = 10), **d** Pancreatic polypeptide (PP) (*n* = 11) and **e** Insulin (*n* = 12). Values represent mean ± 95% CIs. *P < 0.05 versus corresponding time point in other trial. B = Breakfast period, in which participants ingested a prescribed breakfast during the FED trial and remained fasted during the FASTED trial, *E* = Exercise period, where participants completed a 45 min brisk walk, L = Lunch where 400 g chicken and sweetcorn soup was ingested

No main effect of trial, main effect of time or trial × time interaction effect was seen for GHR concentrations (*P* = 0.192, *P* = 0.134, *P* = 0.110, respectively; Fig. 6a) or PYY concentrations (*P* = 0.929, *P* = 0.382, *P* = 0.839, respectively; Fig. 6c).

For GLP-1, there was a main effect of trial (*P* = 0.017) but no main effect of time (*P* = 0.351) or trial × time interaction effect (*P* = 0.104). GLP-1 concentrations were higher in the FED compared with FASTED trial post breakfast at 15 min (5.86 ± 7.26 vs. 3.21 ± 4.98 pmol/l; *P* = 0.014), 1 h post breakfast at 75 min (3.44 ± 4.14 vs. 2.45 ± 3.74 pmol/l; *P* = 0.012) and pre-lunch at 150 min (4.09 ± 6.46 vs. 2.68 ± 5.14 pmol/l; *P* = 0.033) (Fig. 6b).

A main effect of trial (*P* = 0.019), a main effect of time (*P* = 0.008) and a trial × time interaction effect was observed for PP (*P* = 0.012) (Fig. 6d). PP concentrations were higher during FED compared with FASTED pre-exercise at 75 min (23.42 ± 17.57 vs. 10.99 ± 2.76 pmol/l; *P* = 0.036), post exercise at 120 min (95.11 ± 99.70 vs. 29.37 ± 42.73 pmol/l; *P* = 0.016), 30 min post exercise at 150 min (30.96 ± 24.41 vs. 14.60 ± 13.82 pmol/l; *P* = 0.017), and 30 min post soup consumption at 180 min (67.35 ± 44.18 vs. 42.96 ± 27.11 pmol/l; *P* = 0.010).

No main effect of trial (*P* = 0.529) but a main effect of time (*P* < 0.001) and a trial × time interaction effect (*P* < 0.001) was seen for insulin concentrations. Insulin concentrations were higher during FED compared with FASTED immediately post breakfast at 15 min (139.56 ± 86.54 vs. 81.59 ± 55.91 pmol/l; *P* = 0.010) and pre-exercise at 75 min (119.11 ± 65.28 vs. 74.18 ± 58.56 pmol/l; *P* = 0.001). Insulin concentrations increased in the FASTED trial greater than the FED trial 1.5 h post soup consumption at 240 min (86.04 ± 48.18 vs. 69.09 ± 49.29 pmol/l; *P* = 0.006) (Fig. 6e).

## Discussion

Fasted brisk walking did not result in any difference in GER of a subsequent meal compared with brisk walking in the postprandial state. Minimal differences in perception of appetite post exercise were observed. Fasted exercise had limited effects on metabolic and gut hormone responses compared with exercise performed after breakfast ingestion. These findings suggest that fasted low-intensity exercise may not elicit a compensatory effect of energy intake in the immediate hours following exercise.

To the authors knowledge, this is the only study that has investigated GER between fasted vs. fed exercise. A difference in GER between trials may not have been observed in this present study owing to a relatively low meal volume and energy content. Alternatively, no difference may have been seen owing to the high variation in participants BMI and body fat percentages. Studies investigating the influence of BMI on GER are inconsistent, with some studies reporting no differences in GER according to BMI, whereas some have [41–44]. The present study used a soup meal that contained a large liquid component. The transient differences in GLP-1 and PP concentrations prior to lunch may potentially affect the emptying rate of a solid meal instead owing to a greater delay of emptying with solid food compared with liquids [45]. A suppression of appetite and the orexigenic hormone acylated GHR has been consistently reported during and briefly following moderate-to-high intensity bouts of running exercise [24, 29, 30]. However, in the present study, GHR was not suppressed post exercise in either trial. This lack of suppression is consistent with observations by King et al. [26] who also failed to observe an immediate difference in GHR following an acute bout of walking exercise. The reduced physiological challenge imposed by brisk walking compared with running exercise of a higher intensity may account for these conflicting effects of exercise.

Concentrations of the satiety hormone PYY has also been shown to increase with an acute bout of aerobic exercise [27, 29]. However, the present study showed a lack of difference in PYY concentrations over time and between trials, especially post exercise, which coincides with the subsequent hunger and satiety ratings. These results may be owing to the relatively small calorie content of the breakfast provided within the present study (724 kJ (173 kcal)) in comparison with those provided in most other investigations (typically > 1674 kJ (400 kcal)). In addition, total PYY was measured in the present study. The measurement of the active form PYY_3–36_would have been more desirable.

The findings for GLP-1 and PP are in agreement with previous literature regarding elevated levels following food consumption. PP levels remained higher in the FED trial post exercise, which is consistent with literature reporting elevated PP levels following acute exercise performed in the fed state with no effect on GHR levels [46]. Elevated concentrations of GLP-1 and PP are often accompanied by decreased perceptions of hunger [25, 47]. This is reflected within the findings of the present study until post exercise where the hunger scores dissociate from PP as hunger did not change post exercise. A possible explanation for this is that hunger ratings may be influenced more by PYY and GHR concentrations. The combined lack of differences in these hormones and hunger post exercise could suggest that regardless of an increased energy expenditure being incurred from the exercise, there will likely be no compensatory increase in energy intake post exercise to account for the omission of energy intake prior to the exercise. This may, therefore, create a small short-term negative energy balance and if sustained in the long-term, the cumulative effects may have an important role in weight maintenance. However, it is important to consider that although appetite is anticipated to reflect energy intake, the two do not always correlate, and a delayed response in energy intake may still be possible, although some studies have not reported this finding [48, 49]. Further research on both the shorter-term effects of an acute bout of exercise and the cumulative effects of fasted exercise over a period of time is required.

Fasted brisk walking did not alter glycaemic, triglyceride or cholesterol responses to subsequent meal ingestion. However, concentrations of NEFA were greater in the FASTED trial pre- and post exercise, then lower post subsequent meal ingestion. Increased NEFA prior to the lunch meal indicates greater fat mobilisation for metabolism and is consistent with the knowledge that fat oxidation is increased with fasting, although the results of the present study only show a tendency of increased fat oxidation during fasted exercise. This may have been due to the relatively low intensity of exercise performed in this study. However, carbohydrate was the preferred substrate for exercise in this trial rather than fat stores owing to the provision of exogenous carbohydrate from the breakfast consumption.

In conclusion, these findings demonstrate that GER, appetite and appetite regulatory hormones are not sensitive to an acute bout of low-intensity exercise in the fasted state compared with the fed state. The indication that no compensatory increase in energy intake will occur post exercise potentially holds positive implications for fasted brisk walking in the long-term control of body mass. Future research is warranted to investigate the influence of fasted exercise on appetite and appetite regulatory hormones over an extended duration to help further understanding on the influence of fasted exercise on energy balance and metabolic health.
